# Using Generative AI to Extract Structured Information from Free Text Pathology Reports

**DOI:** 10.1007/s10916-025-02167-2

**Published:** 2025-03-13

**Authors:** Fahad Shahid, Min-Huei Hsu, Yung-Chun Chang, Wen-Shan Jian

**Affiliations:** 1 Graduate Institute of Data Science, College of Management, Taipei Medical University, Taipei, Taiwan; 2 Department of Neurosurgery, Shuang-Ho Hospital-Taipei Medical University, Taipei, Taiwan; 3 International Ph.D. Program in Biotech and Healthcare Management, Taipei Medical University, Taipei, Taiwan; 4 School of Healthcare Administration, Taipei Medical University, Taipei, Taiwan; 5 Professional Master Program in Artificial Intelligence in Medicine, Taipei Medical University, Taipei, Taiwan

**Keywords:** Artificial Intelligence, Breast Cancer, ChatGPT, Free-text Pathology Reports, Generative AI, Information Extraction, Large Language Models, Streamlit

## Abstract

Manually converting unstructured text pathology reports into structured pathology reports is very time-consuming and prone to errors. This study demonstrates the transformative potential of generative AI in automating the analysis of free-text pathology reports. Employing the ChatGPT Large Language Model within a Streamlit web application, we automated the extraction and structuring of information from 33 unstructured breast cancer pathology reports from Taipei Medical University Hospital. Achieving a 99.61% accuracy rate, the AI system notably reduced the processing time compared to traditional methods. This not only underscores the efficacy of AI in converting unstructured medical text into structured data but also highlights its potential to enhance the efficiency and reliability of medical text analysis. However, this study is limited to breast cancer pathology reports and was conducted using data obtained from hospitals associated with a single institution. In the future, we plan to expand the scope of this research to include pathology reports for other cancer types incrementally and conduct external validation to further substantiate the robustness and generalizability of the proposed system. Through this technological integration, we aimed to substantiate the capabilities of generative AI in improving both the speed and reliability of data processing. The outcomes of this study affirm that generative AI can significantly transform the handling of pathology reports, promising substantial advancements in biomedical research by facilitating the structured analysis of complex medical data.

## Introduction

Pathology reports are indispensable in the medical diagnostic process, providing crucial insights for disease diagnosis and treatment, particularly in oncology. These reports inform clinical decisions and contribute significantly to epidemiological research, thus impacting patient care and advancing medical knowledge [1]. Despite their importance, the narrative, free-text format of these reports poses significant challenges for data extraction and analysis. The variability and complexity inherent in these reports impede systematic extraction of structured information, which is essential for analyzing large data sets, classifying trends and developing predictive models in healthcare [2]. Traditionally, the extraction and structuring of data from pathology reports have been predominantly manual, making the processes not only time-consuming but also susceptible to errors. These limitations are particularly critical in fields like oncology, where the precision and timeliness of information are paramount for effective patient treatment and research [2].

In recent years, Natural Language Processing (NLP) and Deep Learning (DL) have been leveraged to automate the extraction of data from these reports [3]. Early NLP efforts were largely rule-based and statistical in nature, providing foundational methods for parsing and structuring text [4, 5]. However, as demonstrated in broader studies, these methods have often struggled with capturing the complexities of medical language in fields beyond oncology, as highlighted in recent literature [6]. As NLP evolved, Deep Learning models, particularly those employing convolutional neural networks (CNNs) and deep neural networks (DNNs) began to play a significant role due to their ability to manage unstructured data and learn from vast amounts of training examples. These advanced DL methodologies, while effective, often require extensive and well-annotated datasets which are not only costly but also scarce, as detailed in the study [6] on integrated DL and NLP for continuous remote monitoring, While traditional DL models like BERT have facilitated advancements, their reliance on extensive fine-tuning, which demands labeled data and significant computational resources, limits their practical deployment. In contrast, GPT-3.5 offers a more streamlined approach with OpenAI’s ready-to-use API, simplifying integration into existing systems without the need for extensive infrastructure setup.

Despite these advancements, DL models in pathology report analysis often face challenges related to the need for extensive labeled datasets, which are expensive and time-consuming to create [7]. Additionally, DL models can struggle with the variability of medical terminology and the idiosyncrasies of report formats, which can vary widely across institutions and languages [8, 9]. These challenges highlight the difficulty of applying such models universally in clinical practice, where adaptability and accuracy are crucial [10–12].

BERT utilizes a transformer encoder architecture focused on bidirectional context processing, which, while robust for short texts, often struggles with the coherent generation of long-form content. Conversely, GPT-3.5 employs a transformer decoder architecture, enhancing its capability to generate extensive, contextually rich text, better suiting the complex narratives found in pathology reports.

There is an urgent need for an automated solution that can efficiently structure unstructured pathology reports, enhancing data accuracy and accessibility. This solution must be scalable, capable of adapting to various types of pathology reports and robust enough to support global interoperability. The ability to convert free text into structured data systematically would revolutionize pathological data analysis, enabling more sophisticated research and improved healthcare outcomes worldwide [13].

The introduction of Generative AI, particularly through the use of advanced Large Language Models (LLMs) like GPT, offers a transformative solution to these challenges [14, 15]. Furthermore, GPT-3.5’s versatility allows it to excel across a broad range of NLP tasks without the need for domain-specific tuning, unlike BERT which requires versions like BioBERT for optimized performance in medical contexts. Additionally, GPT-3.5's user-friendly design accepts natural language prompts and delivers direct, fluent responses, significantly enhancing usability compared to BERT’s more complex requirements for input tokenization and output handling. LLMs excel in understanding, interpreting and generating text, which allows for precise extraction and structuring of relevant information from free-text pathology reports [16, 17]. The advanced learning capabilities of GPT-3.5, supporting zero-shot and few-shot learning, empower it to quickly adapt to new tasks, a stark contrast to BERT, which necessitates detailed training with labeled data for most new tasks. This capability marks a significant advancement over previous AI models by standardizing data extraction processes, enhancing the accuracy of data analysis and enabling the integration of data from diverse sources [18, 19].

This study focuses on harnessing Generative AI to extract structured information from breast cancer free-text pathology reports, testing its efficacy in a tightly defined context to provide a robust foundation for broader applications. The research aims to overcome the limitations of prior DL and NLP approaches by demonstrating how Generative AI can revolutionize the analysis of pathological data, thereby enhancing research capabilities and improving patient care globally.

## Materials and Methods

### Data Collection

**Fig. 1:**
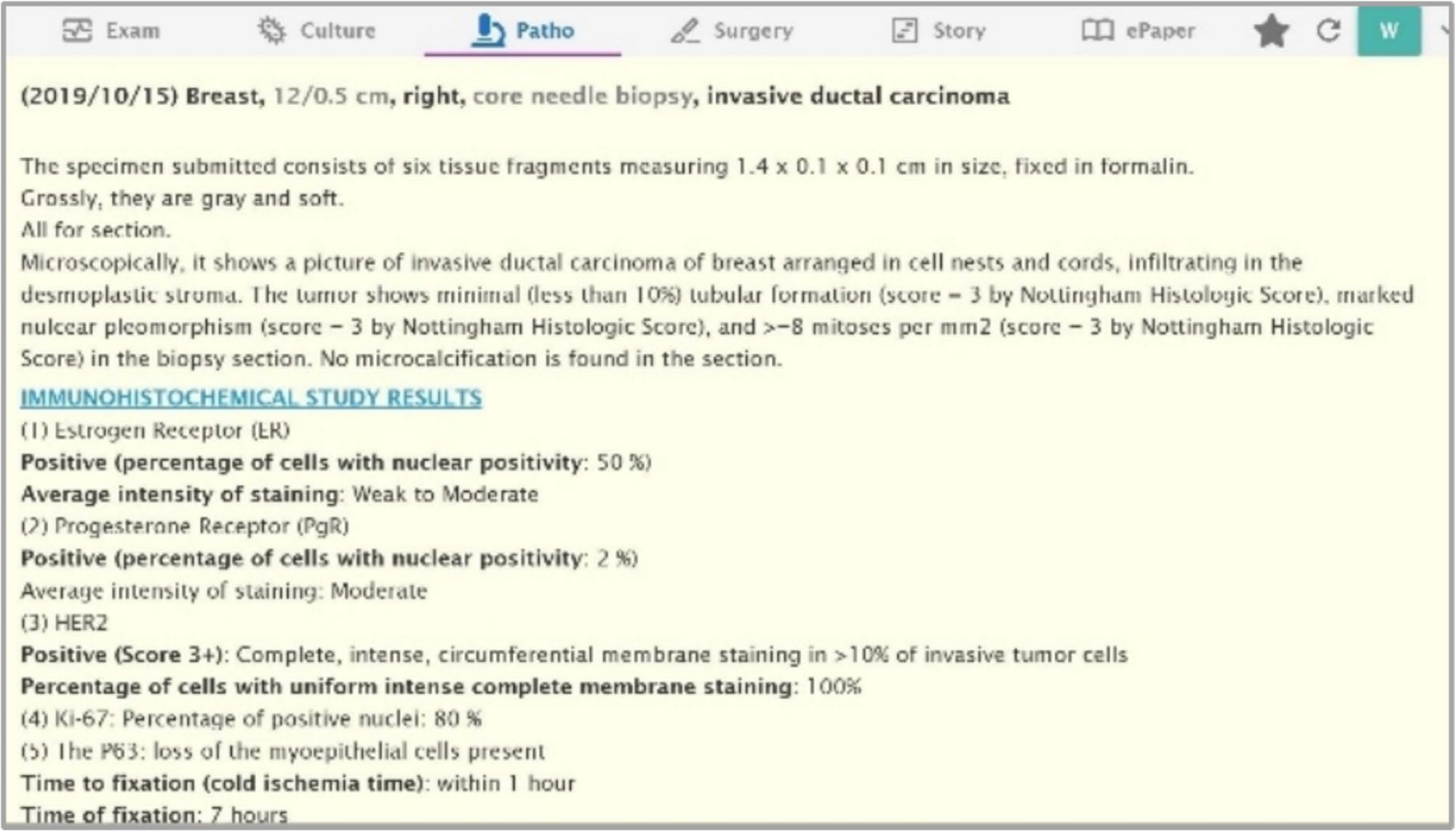
Screenshot demonstrating the free-text format of a pathology report (example from Taipei Medical University)

The dataset comprises 33 anonymized breast cancer free-text pathology reports sourced from Taipei Medical University Hospital as one of them is illustrated in Fig. 1.

These reports, representative of a broad spectrum of breast cancer cases, were chosen to ensure a comprehensive analysis applicable to real-world clinical scenarios. The diversity of the dataset is crucial for the development of a robust AI model capable of accurately structuring and extracting information from free-text pathology reports. We acknowledge the concerns regarding the limited size of our dataset and its potential impact on the generalizability of our findings. To address these issues, we plan to explore opportunities to expand our dataset to include more diverse reports from multiple institutions. This will enable a more robust validation of our AI model’s capabilities across a broader clinical spectrum. Additionally, we will conduct statistical tests to assess the sufficiency of our sample size for capturing the complexity and variability of breast cancer pathology reports. These evaluations will be aligned with established research methodologies and best practices in the field to ensure the reliability and applicability of our findings.

### Research Design and Prototype Development

**Fig. 2:**
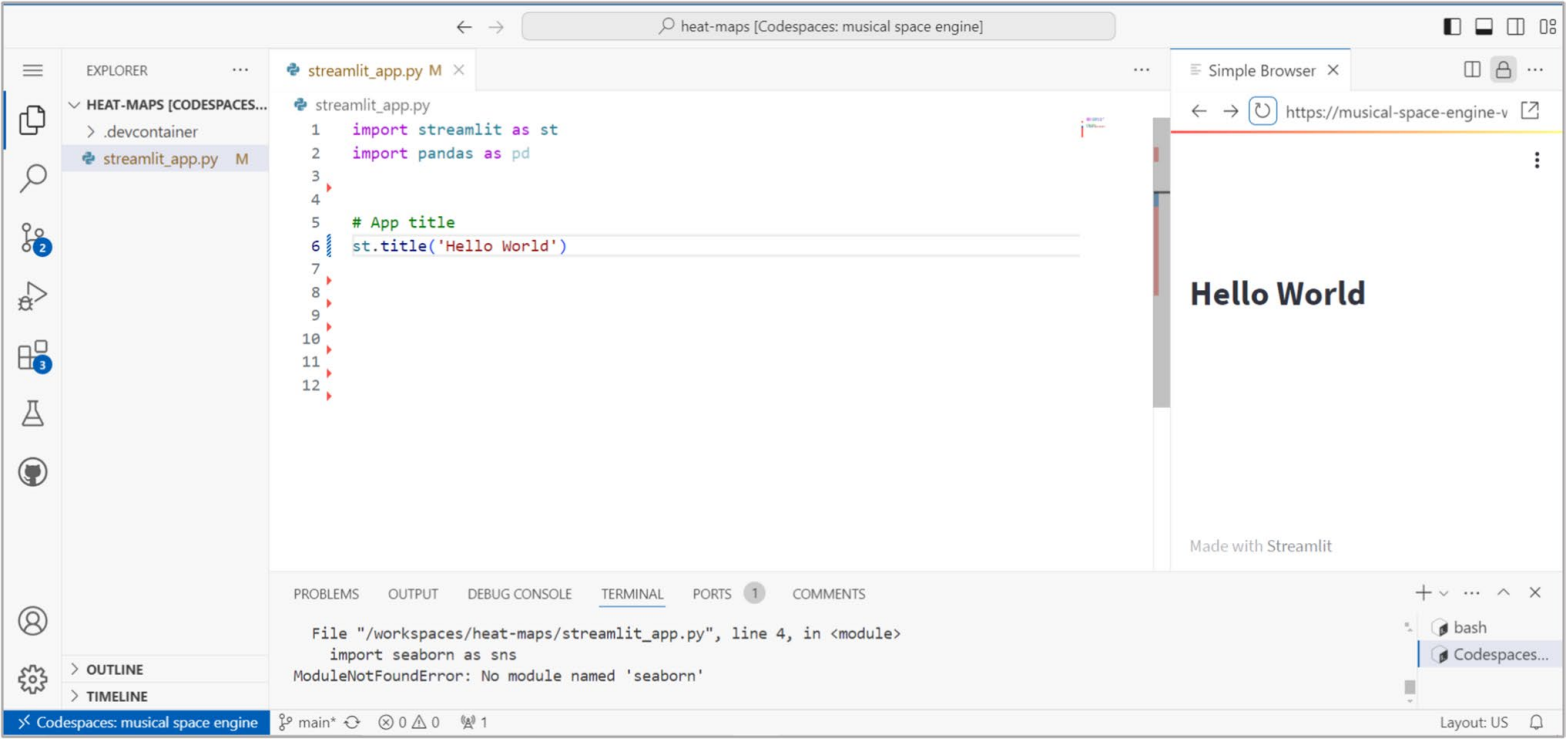
Integrated Development Environment Interface

This study employed a structured approach using Large Language Models (LLMs) to automate the extraction and structuring of information from the pathology reports. The primary algorithm used is the Generative Pre-trained Transformer (GPT), integrated into a custom-built Streamlit.io web application. Streamlit.io, an open-source platform, was chosen for its ability to facilitate rapid development of generative AI applications. This platform allowed for the direct use of our GitHub profile and an integrated development environment (IDE) through GitHub Codespaces, enabling coding in a browser without local IDE installations as showing in Fig. 2. This setup supports coding and deploying directly on Streamlit.io’s servers, streamlining the development process by eliminating the need for traditional deployment pipelines.

### Prototype and Algorithm Integration

**Fig. 3:**
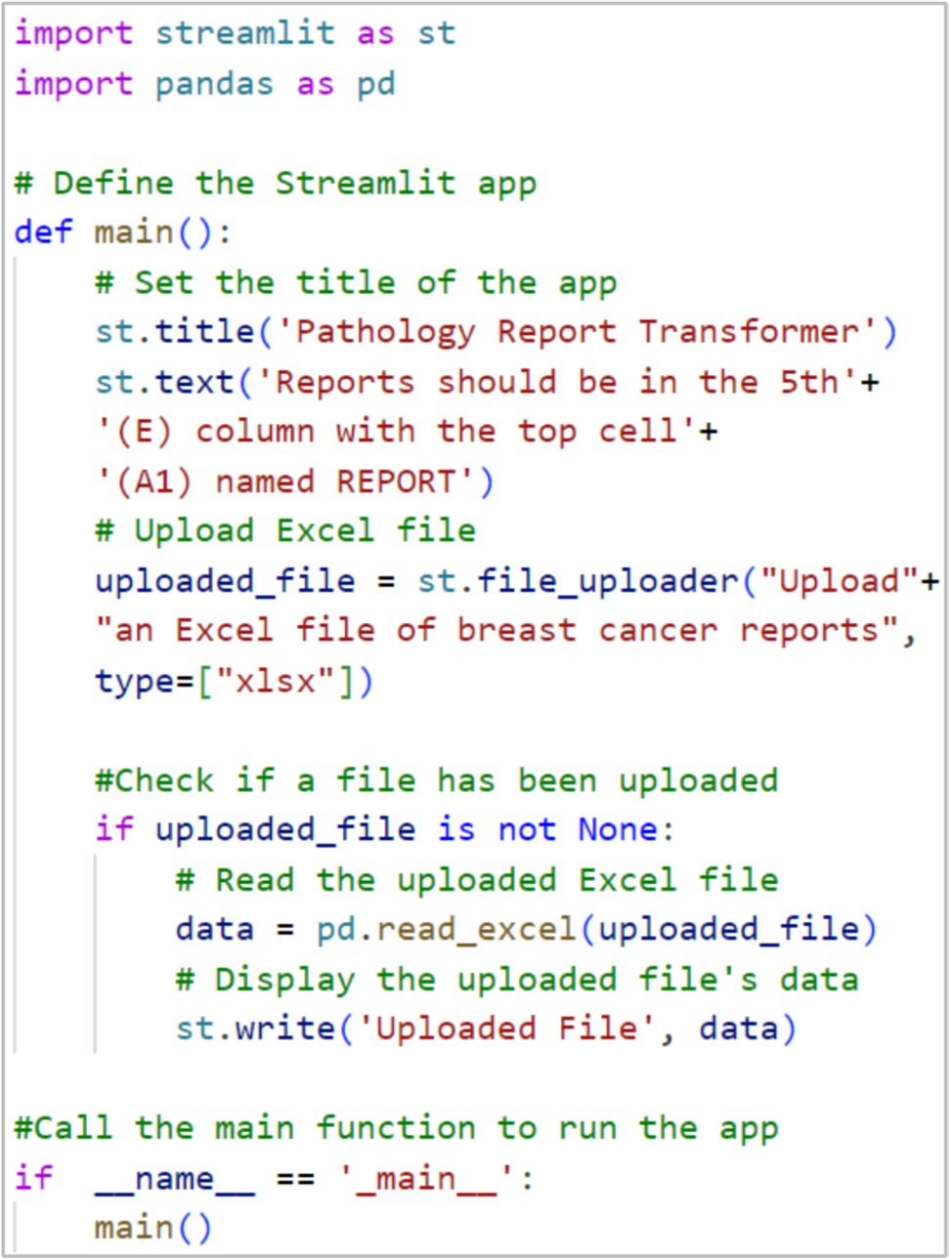
Initial Coding Step

The user interface of the prototype is a straightforward single-page application (SPA), emphasizing simplicity and ease of use. The front-end development began by importing essential libraries like streamlit for app framework and pandas for data manipulation. The main function of the Streamlit app (main) encapsulates all functionalities, including file uploading and data processing controls. An Excel file uploader was implemented to allow users to input their pathology reports, which are then processed into a panda’s DataFrame for data structuring. The code structure is displayed in Fig. 3.

### API Integration and Data Processing

**Fig. 4:**
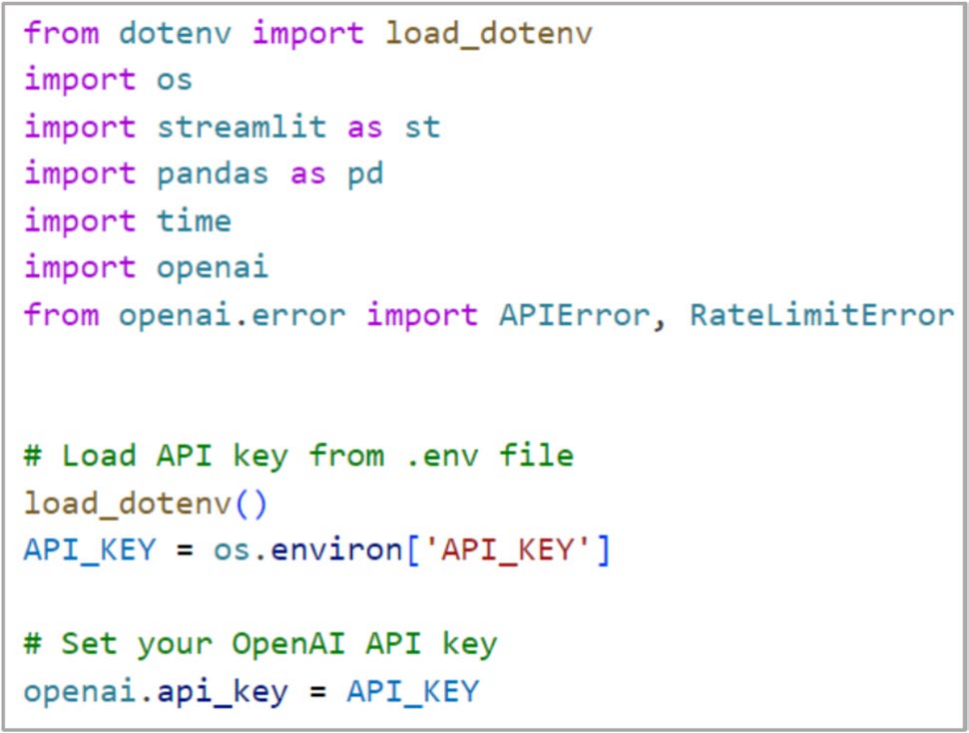
Libraries and API

The selection of GPT-3.5 over other LLMs such as BERT or BioBERT was driven by GPT-3.5's superior performance in understanding and generating complex language patterns, crucial for processing the nuanced language found in medical pathology reports. Comparative studies have shown that GPT-3.5 provides enhanced context capture and coherence in generating textual interpretations, which is vital for the accurate extraction and structuring of data from unstructured medical texts. These capabilities make GPT-3.5 particularly adept at handling the specialized vocabulary and varied syntactical structures prevalent in pathology reports. Integration with the OpenAI API uses the GPT-3.5 model, managed through environment variables and secure API key handling using dotenv as displayed in Fig. 4. Rate limits are carefully managed to ensure reliable API performance, incorporating strategies such as extended pauses between requests to handle API constraints effectively. The application features robust error handling mechanisms to maintain operation continuity and data integrity.

### Prompt Engineering and Data Extraction

The effectiveness of GPT-3.5 in our application is significantly enhanced by meticulous prompt engineering. This process involves the strategic formulation of input prompts to the model to optimize clarity and specificity in the information retrieval process. Through iterative refinements, these prompts are tailored to align closely with the idiosyncratic expressions and terminology of breast cancer pathology, which significantly contributes to the high accuracy rates observed. This method underscores the importance of model tuning and adaptation in leveraging generative AI effectively in specialized domains, which involves designing and refining inputs to the AI model to optimize output accuracy for medical data extraction [10, 20]. This systematic approach is crucial due to the complexities and nuances of medical data. Each prompt is meticulously crafted and iteratively refined based on performance feedback, ensuring high accuracy in extracting breast cancer-specific information according to ICCR protocols [21]. In practice, our system utilizes a loop function to systematically issue these crafted prompts to the ChatGPT model via the OpenAI API. This function iterates over a sequence of structured prompts, each designed to extract specific pieces of medical information from the pathology reports. As each prompt is processed, the AI’s response is evaluated for accuracy and relevance. If the response fails to meet our strict accuracy thresholds or appears incomplete, the prompt is adjusted dynamically and re-submitted. This adaptive prompt engineering process ensures that the extracted information is both precise and comprehensive, adhering to established ICCR protocols for data integrity and reliability. The strategic use of this iterative loop mirrors methodologies discussed in [22] which highlights the importance of tailored prompts in effectively extracting structured medical information using generative AI models [22].

Old Prompts:

prompt = 

f"Check ER, PgR, and HER2 is positive or Not\n\n".

f"Show the result in the following format: \n".

f"ER: Positive/Negative\n".

f"PgR: Positive/Negative\n".

f"HER2: Positive/Negative\n".

f"Identify the laterality of the specimen as well (right, left, or unspecified).\n\n".

f"Laterality: Right/Left/Not specified\n\n".

f"Extract the dimensions of the specimen.\n\n".

f"Dimensions: Width Height Depth \n\n".

f"Tumour Focality: Cannot be assessed/Single focus/Multiple foci\n\n".

f"Determine the histological tumour type. If the tumour type is 'Mixed', specify the subtypes present.

f"Histological Tumour Type: Description\n\n".

f"Assess the histological tumour grade and include any relevant scores or details if the score cannot.

f"Histological Tumour Grade: Description\n\n".

f"Determine if carcinoma in situ is present and mention the type if applicable.\n\n".

f"Carcinoma In Situ: Not identified/Present; if present, mention the type\n\n".

f"Determine the presence and involvement of skin in the tumour extension.\n\n".

New Prompts:

"Size_of_foci": "Search the medical report for any mention of the 'Sizes of individual foci'. If a specific size or range is given, strictly provide that in a short sentence, such as '5 mm' or '3–5 mm'. If no sizes are mentioned, strictly respond with 'not specified’.”,

"tumour_dimensions": "Check the medical report for tumor presence. If there is No residual invasive carcinoma, strictly respond with 'No residual invasive carcinoma'. If only microinvasion ≤ 1mm is mentioned, strictly respond with 'Only microinvasion present (≤ 1 mm)'. Otherwise, strictly respond with 'not specified'.",

"max_dimension_largest_focus": "Search the medical report for the maximum dimension of the largest invasive focus if it is greater than 1 mm. strictly Provide the exact measurement rounded to the nearest mm as a single word. If this information is not available, strictly respond with 'not specified'.",

"tumour_additional_dimensions": "Extract the additional dimensions of the largest invasive focus from the report, presented as 'length x width' in mm. If no additional dimensions are mentioned, strictly respond with 'not specified'.",

"tumour_maxWhole_dimensions": "Determine the maximum dimension of the entire tumor field from the report. strictly Provide this dimension in mm. If the dimension cannot be assessed or is not mentioned, strictly respond with 'not specified'.",

"specify dimensions": "Check the medical report to determine if it states that the tumor dimensions cannot be assessed. If 'Cannot be assessed' is mentioned, strictly respond with 'Cannot be assessed' and include any specific reason given in the report. If the report does not address the assessability of tumor dimensions at all, strictly respond with 'not specified'.",

### User Interface and Output Validation

**Fig. 5:**
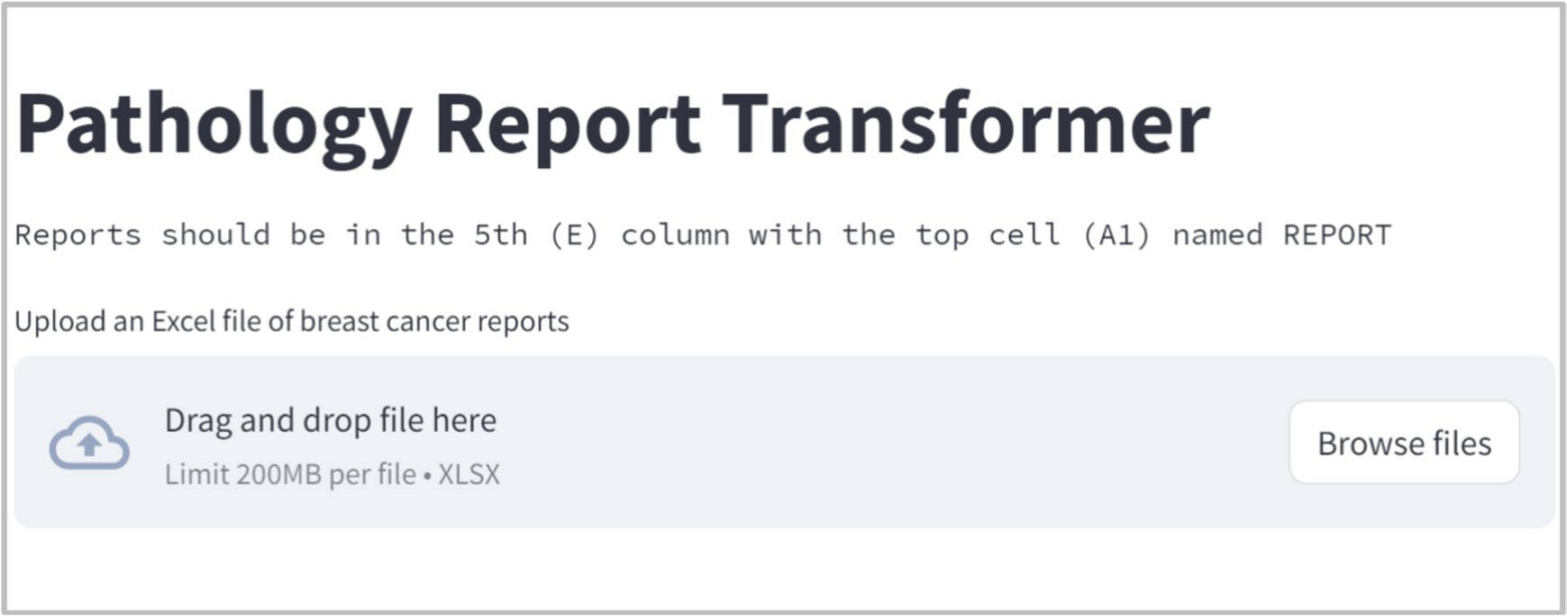
User Interface

**Fig. 6:**
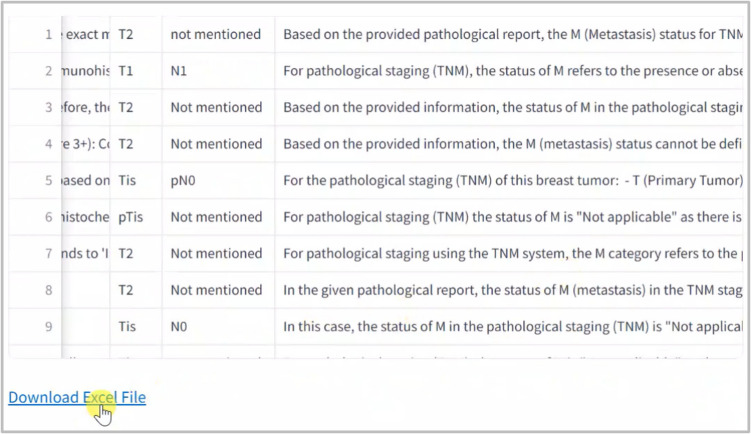
Export Excel File

The Streamlit application serves as the interface where users can upload their pathology reports as shown in Fig. 5, view the extracted data and perform validations. Discrepancies can be corrected directly in the interface, enhancing the utility and accuracy of the application. The validated data is then available for download in Excel format, allowing for further analysis or archival displayed in Fig. 6.

### Overall System Architecture

**Fig. 7:**
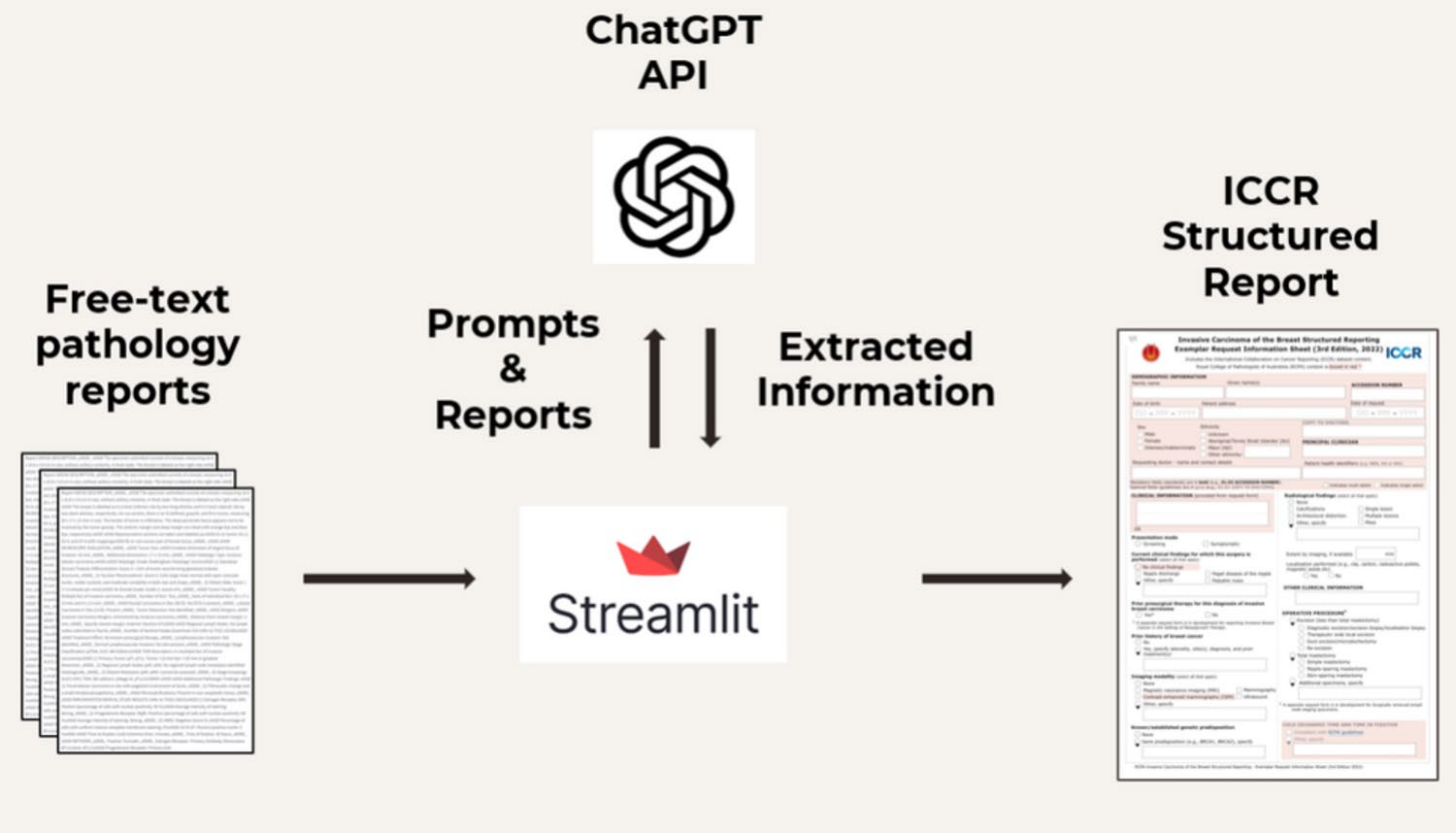
Study framework

**Fig. 8:**
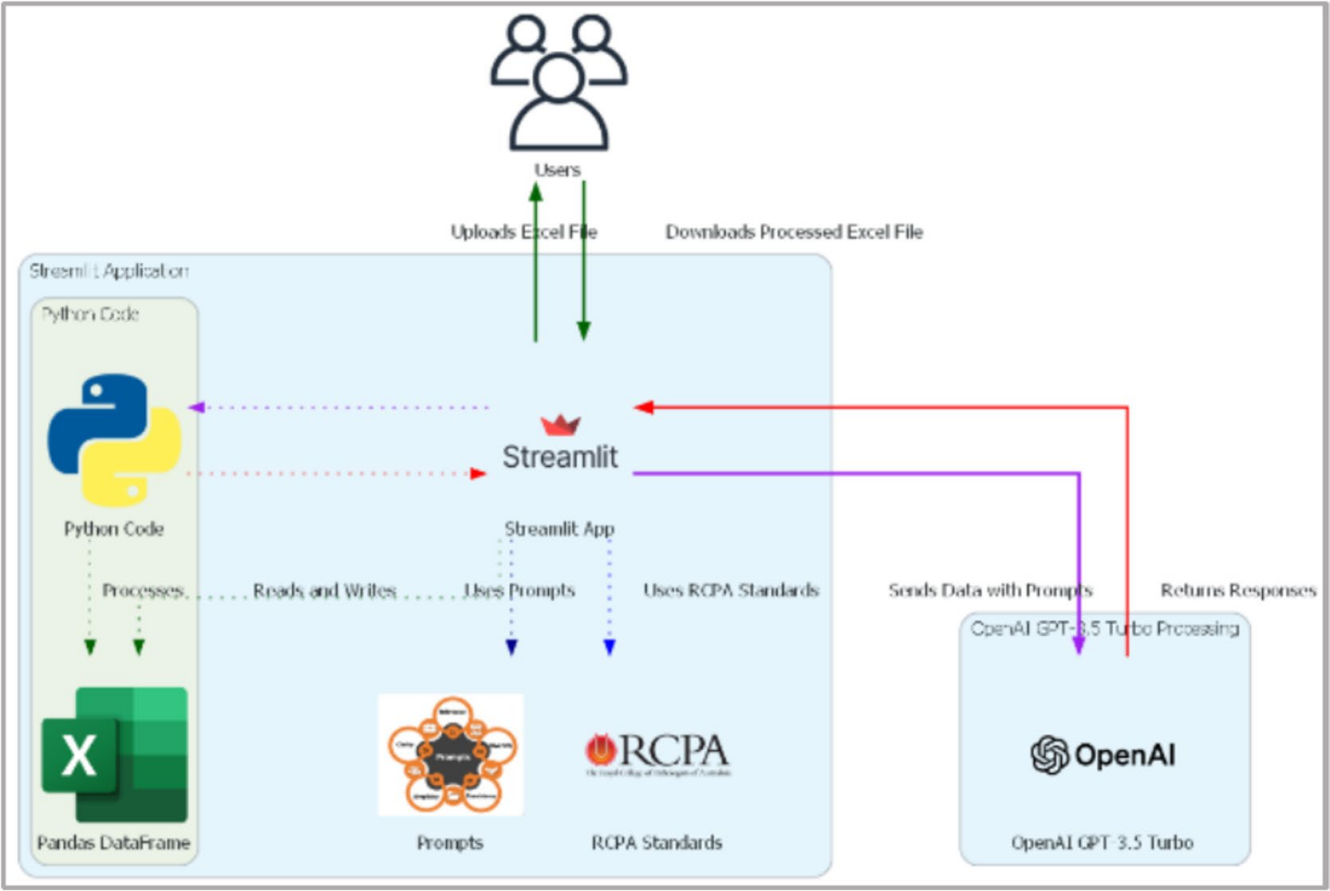
System Architecture

The architecture of the system facilitates a seamless flow from data input through processing to output shown in Fig. 7. The integration of Streamlit, GitHub Codespaces and the OpenAI API forms a robust framework that supports the extraction and structuring of data from pathology reports, transforming unstructured text into structured, analyzable formats. Figure 8 shows the system architecture not only demonstrates the application's functionality but also its potential for scalability and adaptation to other types of medical data analysis.

This methodological framework leverages advanced AI within a user-centric prototype to transform pathology report processing, enhancing accuracy, efficiency and interoperability in medical data analysis.

## Results

In this study, we utilized a generative AI prototype algorithm to successfully extract four major types of information from free-text pathology reports: Macroscopic Information, Microscopic Information, Ancillary Studies and Pathological Staging Information. The prototype, integrating the GPT model within a Streamlit web application, demonstrated its capability to accurately parse and structure complex pathology data from free-text reports.

### Macroscopic Information Extraction

**Fig. 9:**
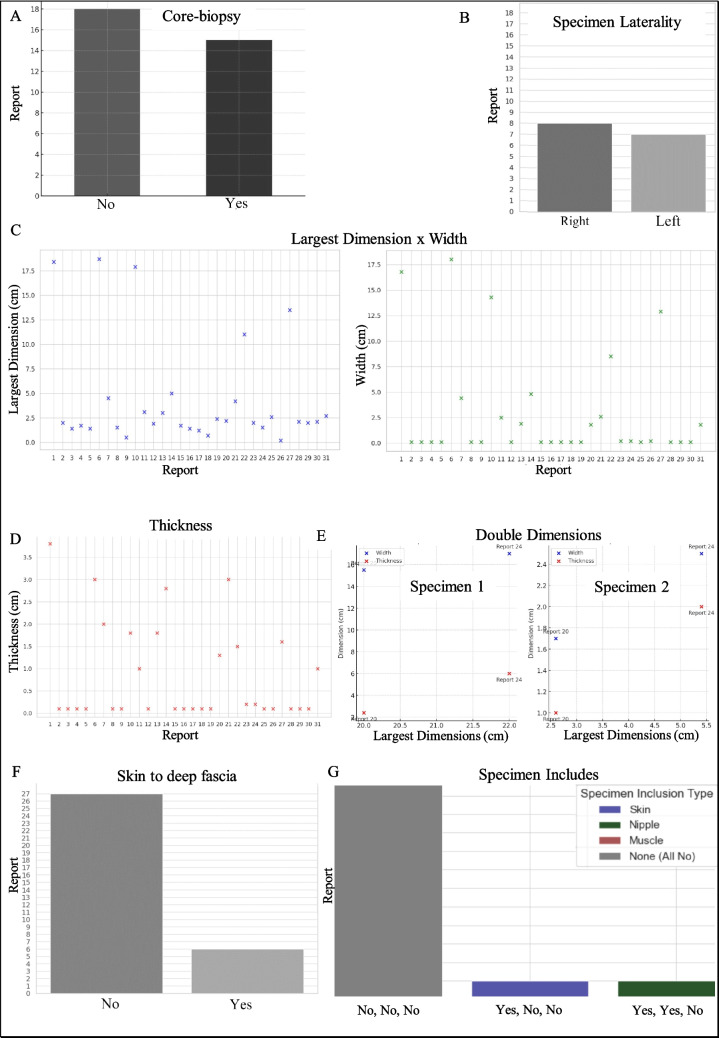
Core biopsy or not core biopsy (**A**). Distribution of core biopsy specimen laterality in reports, categorized as Right or Left (**B**). Measurements of specimen length and width across reports **(C)**. Recorded specimen thickness in reports (**D**). Three-dimensional measurements (length, width, thickness) of two specimens (**E**). Presence of deep fascia in biopsy specimens indicated in reports (**F**). Tissue composition in specimens, showing inclusion of skin, muscle, nipple, or none (**G**)

**Fig. 10:**
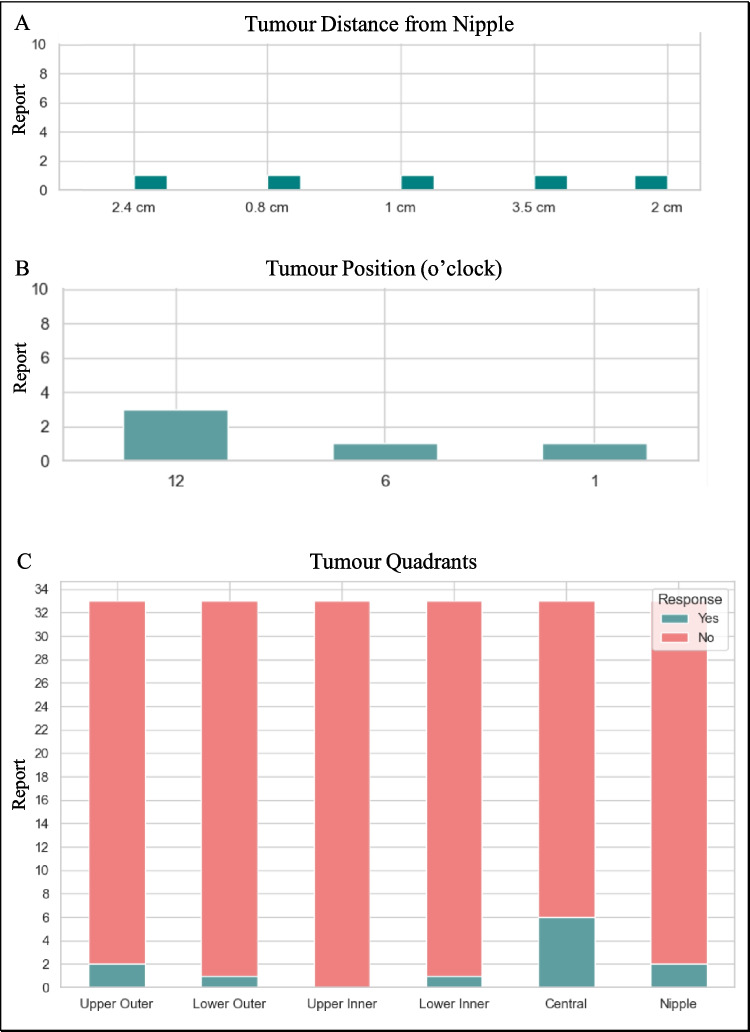
Distribution of tumor distances from the nipple in reports (**A**). Frequency of tumor positions noted in reports, using a clock face orientation (**B**). Distribution of tumors across breast quadrants in reports, with responses categorized as Yes or No (**C**)

The extraction of Macroscopic Information involved analyzing Specimen Laterality and Tumor Site. For Specimen Laterality, out of 33 reports, 15 were identified as core biopsy reports. We determined the laterality for each, with 8 specimens from the right breast and 7 from the left. Our analysis included assessing specimen dimensions and tissue composition, including skin, nipple and skeletal muscle presence. These detailed findings are crucial for understanding the pathological context of each specimen and extracted data is visualized in Figs. 9 and 10.

### Microscopic Information Extraction

**Fig. 11:**
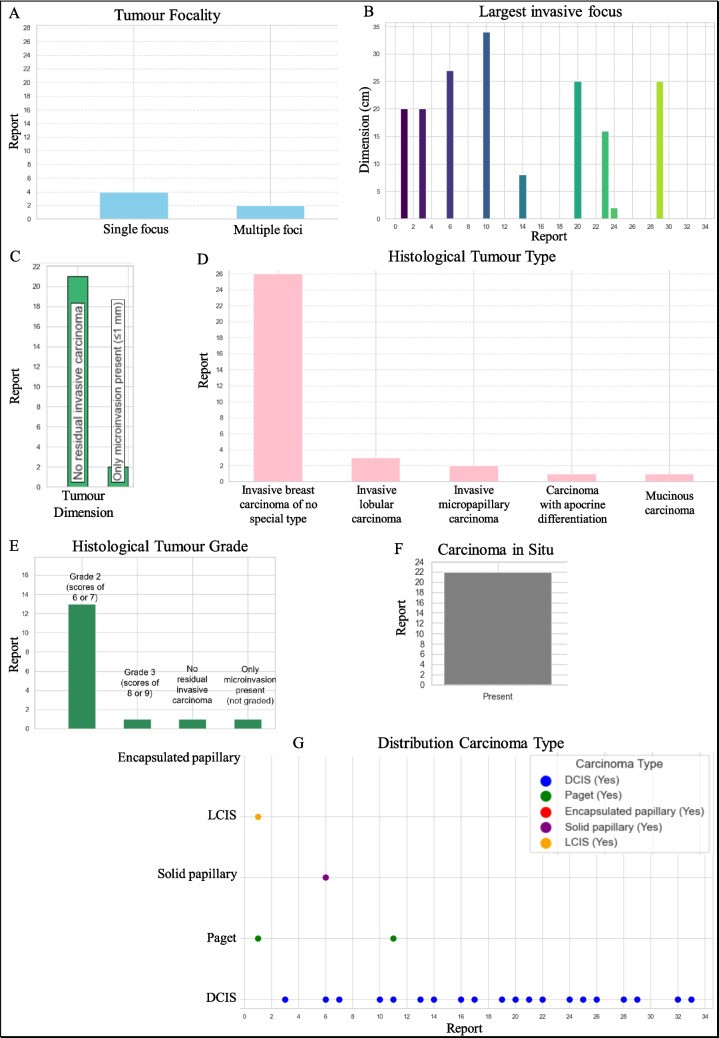
Comparison of reports based on tumor focality: single focus vs. multiple foci (**A**). Distribution of the largest invasive focus dimension reported in centimeters (**B**). Bar graph displaying the most frequently reported tumor dimensions (**C**). Frequency of different histological tumor types mentioned in reports (**D**). Distribution of tumor grades based on their histological scores (**E**). Presence of carcinoma in situ as reported in pathology findings (**F**). Scatter plot showing the distribution of various carcinoma types across reports, categorized by type and presence (**G**)

**Fig. 12:**
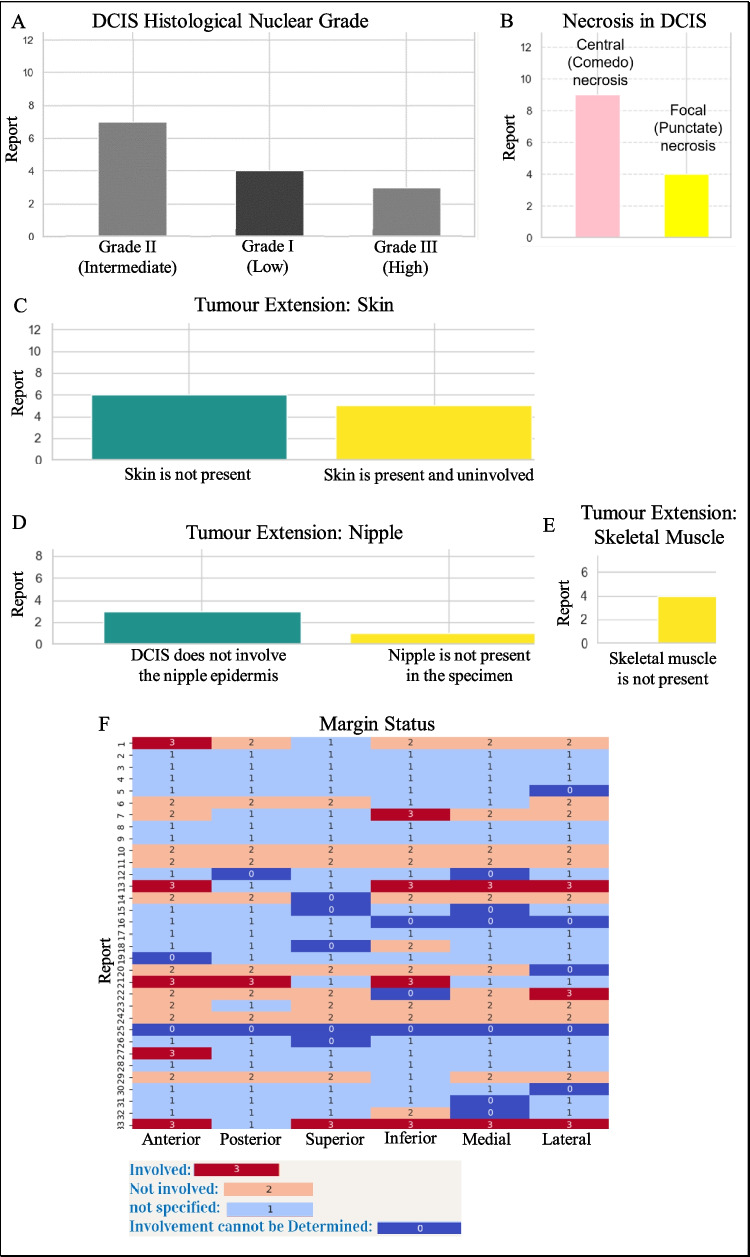
Distribution of DCIS (Ductal Carcinoma in Situ) nuclear grades across reports (**A**). Frequency of central (comedo) and focal (punctate) necrosis in DCIS cases (**B**). Comparison of reports indicating whether the skin is involved or not in tumor extension (**C**). Frequency of nipple involvement or absence in tumor extension cases (**D**). Presence or absence of skeletal muscle involvement in tumor extension (**E**). Heatmap showing the involvement status of tumor margins (anterior, posterior, superior, inferior, medial, lateral) in surgical reports (**F**)

Microscopic Information provided insights into tumor focality, dimensions, histological type and grade. We meticulously extracted data on single versus multiple tumor foci, the size of the largest tumor focus and detailed histological classifications, which are pivotal for diagnostic and treatment decisions. Notably, errors in extraction, such as misinterpretations of tumor distance and position from a manual review, underscored the challenges of parsing complex medical texts. These findings were complemented by visual representations in Figs. 11 and 12, enhancing our understanding of tumor characteristics.

### Ancillary Studies

**Fig. 13:**
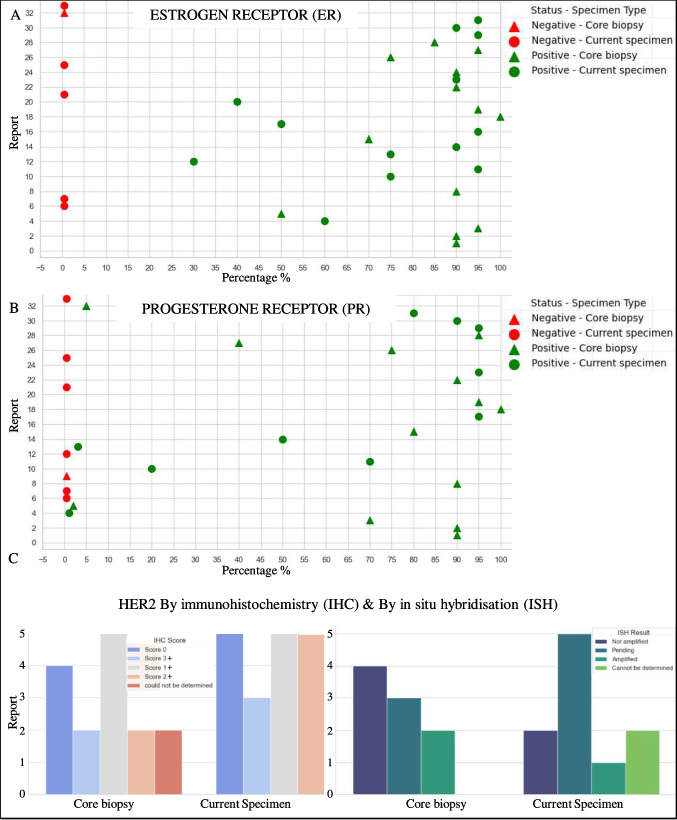
Scatter plot showing the percentage of ER positivity or negativity in core biopsy and current specimen types (**A**). Scatter plot depicting the percentage of PR positivity or negativity in core biopsy and current specimens (**B**). Bar graphs displaying HER2 IHC scores by specimen type and comparing HER2 IHC scores with ISH results (**C**)

The extraction of Ancillary Studies focused on hormone and protein receptor statuses crucial for treatment planning. This included detailed assessments of Estrogen Receptor (ER), Progesterone Receptor (PR) and HER2 statuses displayed in Fig. 13, which are fundamental biomarkers in breast cancer. The accuracy of these extractions was verified through manual reviews, ensuring the reliability of the data for clinical use.

### Pathological Staging Information

**Fig. 14:**
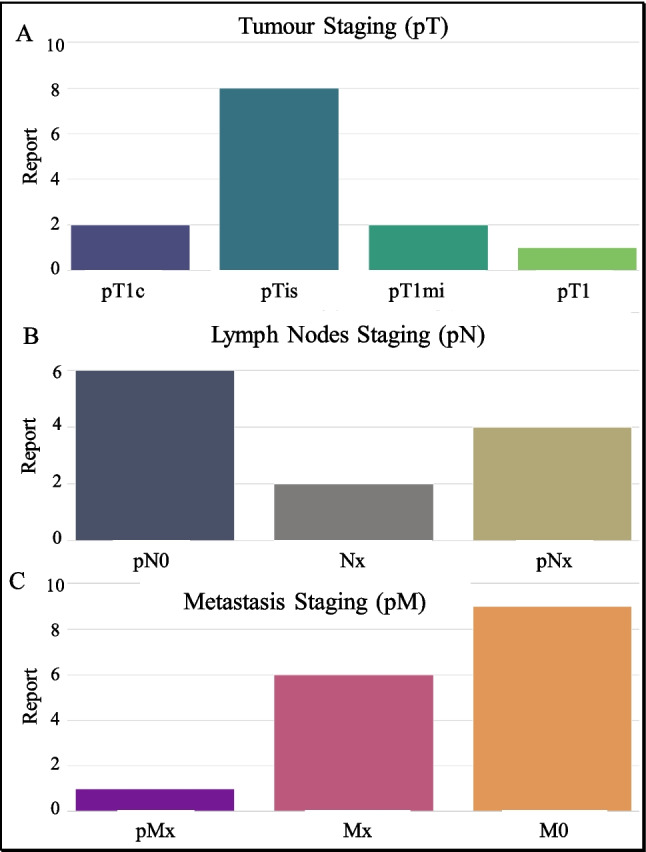
Bar graph showing the distribution of primary tumor staging categories in reports (**A**). Bar graph illustrating the distribution of lymph node involvement stages in pathology reports (**B**). Bar graph depicting the distribution of metastasis stages in pathology reports (**C**)

Our AI prototype effectively extracted comprehensive pathological staging information, including the size and extent of primary tumors (pT), regional lymph node involvement (pN) and distant metastasis (pM) shown in Fig. 14. This staging is critical for treatment strategy decisions and was accurately categorized, reflecting the high capability of the AI system in handling complex staging data.

### Accuracy Assessment

**Fig. 15:**
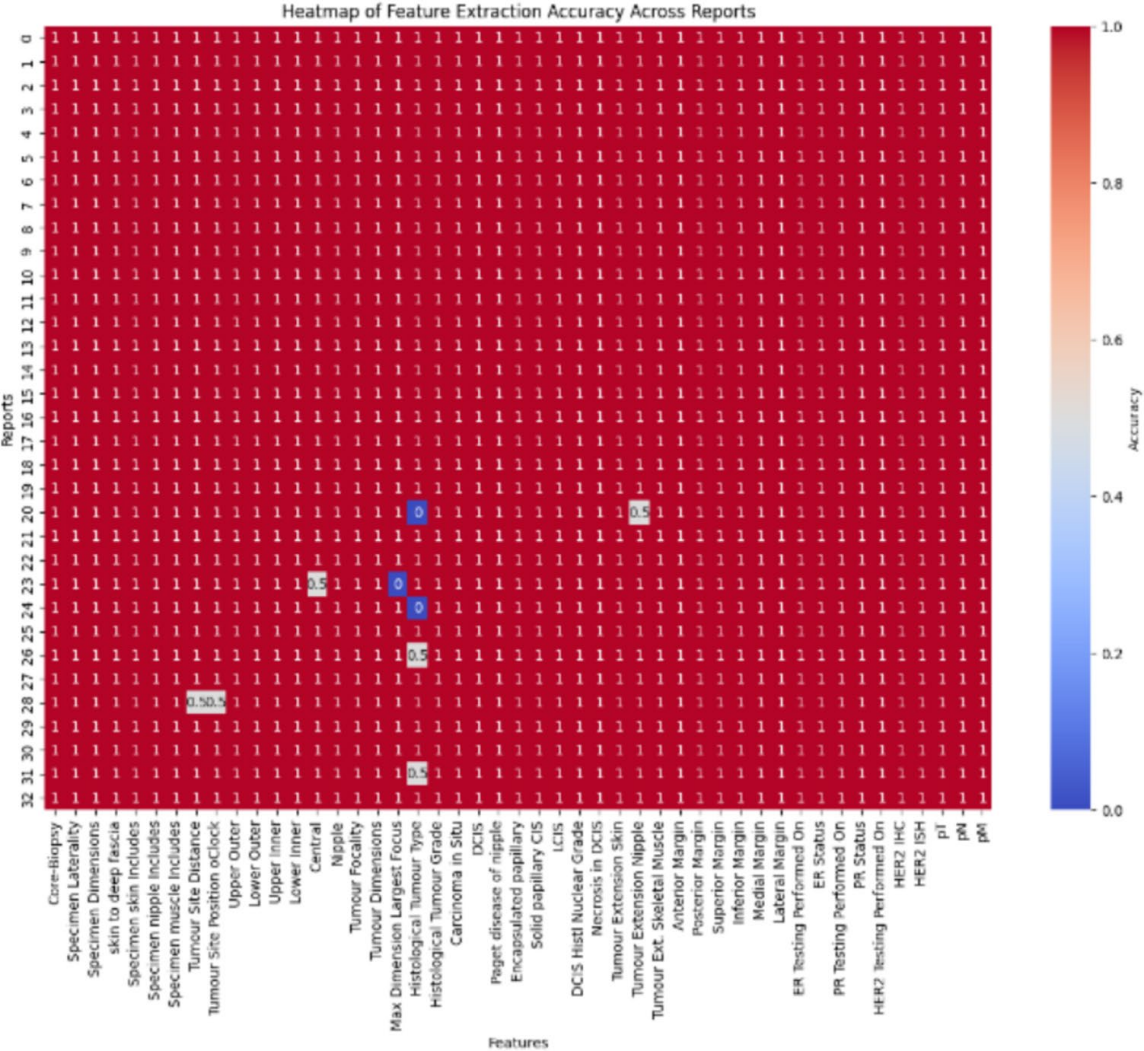
Heatmap of Feature Extraction Accuracy for 47 Pathology Features Across 33 Reports

The overall extraction accuracy was evaluated rigorously, with a manual review process by specialists in breast cancer pathology confirming the reliability of the AI-generated data. The AI prototype achieved an accuracy rate of 99.61%, demonstrating its effectiveness in processing and structuring complex pathological data. Figure 15 displays the accuracy heat map. These results, facilitated by the generative AI prototype, underscore the transformative potential of AI in the digital pathology landscape.

Further analysis of the AI extraction system's performance revealed several areas where refinements could potentially elevate the system's accuracy to 100%. Ambiguous descriptors such as "12/2 cm away from the nipple" led to misinterpretations due to unclear nomenclature, possibly being misconstrued as a fraction rather than intended positional information. Additionally, expressions like "The tumor is located at the upper central portion" posed classification challenges, as 'upper' and 'central' were treated as distinct categories, creating confusion. Moreover, the system's restricted selection for outputs resulted in misclassifications, such as labeling 'adenocarcinoma' as 'Invasive breast carcinoma of no special type' and similar mismatches in terminology like 'micropapillary and cribriform.' Instances where accurately extracted terms, such as 'DCIS does not involve the nipple epidermis,' were not selected in the output further highlight the necessity for an expanded, unrestricted output range. Addressing these issues by refining the system's interpretation and broadening the output parameters will pave the way for achieving a flawless 100% accuracy rate in structuring pathology reports.

## Discussion

The findings of this study emphasize the substantial capabilities of generative AI, particularly the integration of a GPT model with a Streamlit web application, in parsing and structuring complex free-text pathology reports related to breast cancer. This utilization of AI not only refines the extraction of data but also demonstrates a robust method for handling diverse pathology data types, from macroscopic to microscopic details, ancillary studies and accurate pathological staging. Initially, the study utilized the Text-Davinci-003 model, progressing through iterative enhancements to adopt the GPT-3.5 Turbo-0125 model due to its advanced capabilities in generating structured sentence responses. The transition was guided by a necessity for higher accuracy and the obsolescence of earlier models. The integration of Regex was initially considered to refine data extraction but was ultimately abandoned to avoid complications from merging different technological functions. Throughout the study, we faced practical challenges such as managing API rate limits and optimizing cost efficiency, which were crucial in sustaining the functionality of the AI systems used. These operational challenges highlighted the importance of choosing the right model and refining extraction methodologies to balance performance and cost. The adoption of a structured approach to prompt design significantly improved the precision of data extraction. This process involved choosing appropriate models, designing tailored prompts and refining response strategies to ensure the outputs met the required standards for clinical and research applications. Moreover, the study provided insights into practical and procedural elements of pathology, such as the differentiation between core biopsies and mastectomy specimens and the importance of detailed tumor documentation. These aspects are critical for accurate clinical assessments and enhancing the understanding of tumor characteristics. However, the study still has many limitations. Our study only utilized clinical records from a single healthcare system, lacking validation from external institutions. The specificity to breast cancer pathology reports may restrict the generalizability of the findings to other types of cancer or medical conditions. Additionally, while the AI demonstrated high accuracy, the small margin of error observed could still be clinically significant, underscoring the need for continuous refinement.

The integration of AI in pathology has profound implications for the field. It offers the potential to significantly enhance diagnostic efficiency, reduce the workload on pathologists and provide a reliable supplementary tool for clinical decision-making. Future research should focus on expanding the application of this AI model to other types of electronic health records and assessing its performance in real-time clinical settings.

This could pave the way for broader clinical adoption and further innovations in digital pathology.

## Ethical Implications and Integration into Clinical Practice

In compliance with ethical and legal regulations, this retrospective study utilized de-identified, text-based clinical records obtained with informed consent.

The integration of artificial intelligence (AI) into clinical medicine presents significant ethical considerations that must be rigorously addressed. Ensuring the ethical deployment of AI systems involves validating AI models for accuracy and bias, ensuring transparency in AI processes, and adhering to medical ethical standards to prevent misdiagnosis and guarantee equitable patient care.

To effectively integrate AI into real-world clinical settings, it is essential to align these systems with existing clinical workflows, develop intuitive interfaces for clinicians, and provide comprehensive training on the capabilities and limitations of AI.

Establishing robust mechanisms for continuous monitoring and feedback will enhance trust in AI tools and facilitate their adoption, complementing traditional diagnostic methods. Moreover, safeguarding against errors that might lead to adverse patient outcomes is paramount. This necessitates implementing comprehensive validation processes, regular audits, updates based on the latest clinical guidelines, and adherence to healthcare regulations such as HIPAA or GDPR to maintain patient confidentiality.

Engaging with a multidisciplinary team including clinicians, ethicists, and legal experts will ensure that AI deployment aligns with the goals of improving patient care while respecting ethical norms and regulations.

## Directions for Future Research

- **Expanding Data Sets:** Further studies should explore the integration of this AI model with other types of electronic health records to assess its adaptability and effectiveness across different clinical contexts.

- **External validations:** Incorporating multi-institutional data could enhance the model's robustness and generalizability, providing a more comprehensive validation of its utility.
- **Standardizing Data Format:** Adopting international standards, such as those CDM, HL7 and SNOMED, is essential for ensuring data interoperability and facilitating further research.

## Conclusion

This study demonstrates the potential of generative AI in extracting and structuring data from free-text breast cancer pathology reports. By integrating a GPT model with a Streamlit web application, we achieved a 99.61% accuracy rate in data processing, significantly outperforming traditional NLP methods. However, this exceptionally high accuracy is largely attributed to the study’s exclusive focus on breast cancer pathology reports, which inherently follow certain narrative patterns and include most elements required for structured reporting.

We believe that large language models (LLMs) can streamline the structuring of unstructured pathology reports while improving data accessibility and interoperability, ultimately advancing medical research. Future studies should expand the dataset to encompass a broader range of medical conditions and explore the integration of multimodal AI models to further validate and refine the approach.

## Data Availability

No datasets were generated or analysed during the current study.
